# Cytomegalovirus infection and outcome in immunocompetent patients in the intensive care unit: a systematic review and meta-analysis

**DOI:** 10.1186/s12879-018-3195-5

**Published:** 2018-06-28

**Authors:** Xi Li, Yongbo Huang, Zhiheng Xu, Rong Zhang, Xiaoqing Liu, Yimin Li, Pu Mao

**Affiliations:** 1 State Key Laboratory of Respiratory Disease, National Clinical Research Center for Respiratory Disease, Guangzhou Institute of Respiratory Disease, the First Affiliated Hospital of Guangzhou Medical University (Guangzhou Medical University), Guangzhou, China; 2 Intensive Care Unit, The First Affiliated Hospital of Guangzhou Medical University, Guangzhou, Guangdong China; 3 Department of Infection Control, The First Affiliated Hospital of Guangzhou Medical University, Guangzhou, Guangdong China

**Keywords:** Cytomegalovirus, Immunocompetent, Meta-analysis

## Abstract

**Background:**

Cytomegalovirus (CMV) infection is common in immunocompetent patients in intensive care units (ICUs). However, whether CMV infection or CMV reactivation contributes to mortality of immunocompetent patients remains unclear.

**Methods:**

A literature search was conducted for relevant studies published before May 30, 2016. Studies reporting on CMV infection in immunocompetent patients in ICUs and containing 2 × 2 tables on CMV results and all-cause mortality were included.

**Results:**

Eighteen studies involving 2398 immunocompetent patients admitted to ICUs were included in the meta-analysis. The overall rate of CMV infection was 27% (95%CI 22–34%, I^2^ = 89%, *n* = 2398) and the CMV reactivation was 31% (95%CI 24–39%, I^2^ = 74%, *n* = 666). The odds ratio (OR) for all-cause mortality among patients with CMV infection, compared with those without infection, was 2.16 (95%CI 1.70–2.74, I^2^ = 10%, *n* = 2239). Moreover, upon exclusion of studies in which antiviral treatment was possibly or definitely provided to some patients, the association of mortality rate with CMV infection was also statistically significant (OR: 1.69, 95%CI 1.01–2.83, I^2^ = 37%, *n* = 912,). For CMV seropositive patients, the OR for mortality in patients with CMV reactivation as compared with patients without CMV reactivation was 1.72 (95%CI 1.04–2.85, I^2^ = 29%, *n* = 664). Patients with CMV infection required significantly longer mechanical ventilation (mean difference (MD): 9 days (95% CI 5–14, I^2^ = 81%, *n* = 875)) and longer duration of ICU stay (MD: 12 days (95% CI 7–17, I^2^ = 70%, *n* = 949)) than patients without CMV infection. When analysis was limited to detection in blood, CMV infection without antiviral drug treatment or reactivation was not significantly associated with higher mortality (OR: 1.69, 95%CI 0.81–3.54, I^2^ = 52%, *n* = 722; OR: 1.49, I^2^ = 63%, *n* = 469).

**Conclusion:**

Critically ill patients without immunosuppression admitted to ICUs show a high rate of CMV infection. CMV infection during the natural unaltered course or reactivation in critically ill patients is associated with increased mortality, but have no effect on mortality when CMV in blood. More studies are needed to clarify the impact of CMV infection on clinical outcomes in those patients.

**Electronic supplementary material:**

The online version of this article (10.1186/s12879-018-3195-5) contains supplementary material, which is available to authorized users.

## Background

Human cytomegalovirus (CMV) is a prototypic member of the β herpes virus subfamily [1]. The prevalence of CMV seropositivity in human populations is roughly 50–95% [2–4] and highest amongst older people [5]. Cytomegalovirus infection induces innate immune responses (eg. natural killer cells) and adaptive immunity (eg. CD4+/CD8+ T cells). However, the virus can evade host detection by expressing genes that interfere with both the innate and adaptive immune systems. Eventually, CMV is able to establish latency in which either the host fails to eliminate the virus or the virus cannot replicate. However, CMV can become reactivated during periods of host immune suppression [6].

It is well known that CMV infection is common in canonical immunodeficiency patients, such as those with human immunodeficiency virus infection, solid organ or stem cell transplantation and patients undergoing chemo- or radiotherapy [7–9]. With the development of more sensitive detection method, the rate of CMV detection is high in intensive care units (ICUs) [10–25]. However, so far, there is no convincing research to support the use of antiviral treatment when critically ill but immunocompetent patients present with CMV infection. Furthermore, whether CMV is a contributor or simply a bystander to the severity of illness remains under debate [26–28].

Whether CMV infection is associated with increased mortality in immunocompetent ICU patients remains controversial [13–16]. A previous meta-analysis published in 2009 demonstrated that CMV infection was associated with a higher mortality rate, nearly twice that observed in patients without CMV infection [29]. However, this study did not consider the influence of antiviral drugs on clinical outcomes. Moreover, many clinical studies about CMV have been reported in recent years. Thus, to acquire a better understanding of the potential role of CMV infection in contributing to mortality in critically ill patients, especially those not receiving antiviral agents and CMV detected in blood, we performed a meta-analysis of data available in the literature, focusing on the outcome in immunocompetent ICU patients with CMV infection.

## Methods

### Search strategy

A literature search for relevant publications included within the electronic databases PubMed, EMBASE and the Cochrane Library was performed using combinations of the keywords “cytomegaloviruses”, “salivary gland viruses”, “herpes virus”, “cytomegaloviral infection”, “HHV5”, “intensive care”, “critical care”, “critical illness”, “mechanical ventilation”, and “pulmonary ventilator”. All searches were updated on May 30, 2016. No language restriction was enforced. We also consulted relevant reference articles and searched using Google Scholar.

### Study selection

Two researchers (LX and HYB) performed data extraction independently, and any discrepancies were addressed by discussion and reevaluation until consensus was achieved. Observational studies were eligible if they reported on CMV infection in immunocompetent patients in the ICU, and if a 2 × 2 table could be constructed based on CMV results and all-cause mortality. All patients were over 18 years of age. The systematic review included only studies in which all patients were tested for CMV. An episode of CMV infection was defined by one of the examination CMV viral culture, polymerase chain reaction (PCR), CMV antigen (pp65) in blood, tracheal aspirates, urine, or a combination of these. A case was defined by the presence of reactivation, where the patient had CMV infection and was seropositive. Immunocompetent patients were defined as those patients who did not receive a solid organ or hematopoietic stem cell transplant, did not receive immunosuppressive treatment, did not have human immunodeficiency virus infection, did not have primary immunodeficiency, and did not receive chemotherapy or radiotherapy before ICU admission.

### Data extraction and quality appraisal

We obtained information on basic study characteristics (author, year of publication, country of origin, study period, setting, and study design), characteristic population, the site and detection method of sample, CMV seropositivity, CMV infection incidence, all-cause mortality, length of ICU/hospital stay, length of mechanical ventilation, and administration of antiviral drugs.

The Newcastle–Ottawa scale, developed for evaluating the quality of observational studies (Additional file 1: Table S1) [30], was used to assess the validity of included studies.

### Data analysis

Continuous variables are reported as mean or median values and categorical variables are reported as n (%). Meta-analytic pooling was performed for outcome variables with a Logit transformation approach, reporting results as summary point estimates (95% confidence interval, CI). We used the Mantel–Haenszel method to obtain odds ratios (ORs) and 95% CI. When only the median, range, or interquartile range of length of mechanical ventilation and the length of ICU stay were reported, we used simple formulas to estimate the mean and standard deviation [31].

Between-study heterogeneity was examined using the I^2^ measure of inconsistency and the chi-square test of heterogeneity.

To evaluate publication bias, we constructed a funnel plot and used the Egger test. Sensitivity analyses of the Begg’s test were additionally conducted to ascertain the robustness of our findings. All meta-analyses were performed with R software (version 3.3.3 for Windows) and SPSS 18 (IBM, Armonk, NY, USA).

## Results

### Study selection

**Fig. 1:**
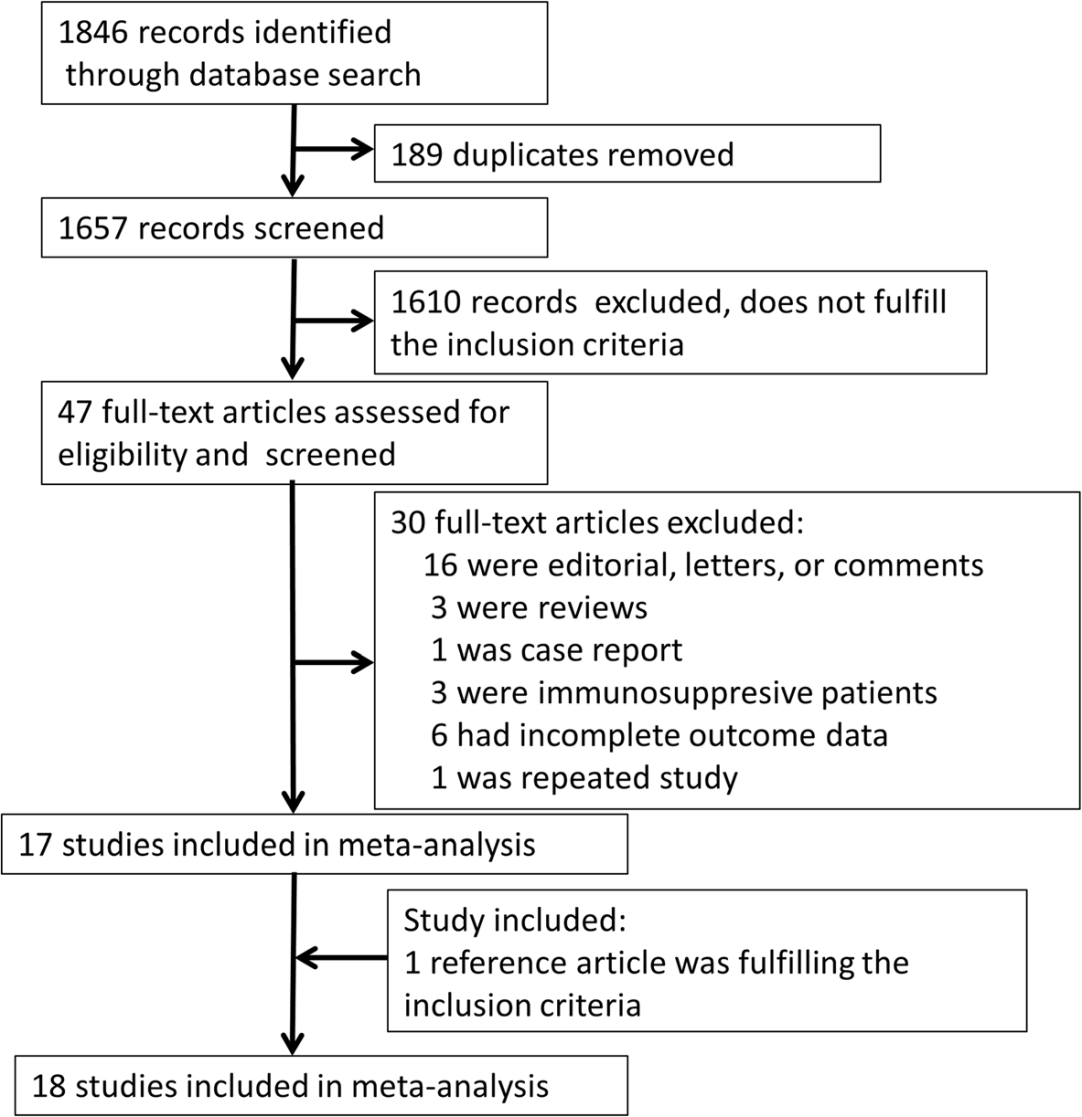
Flow of information through the different phases of a systematic review

The initial database search identified 1846 potentially relevant studies. Following this, assessment of the full text yielded 17 studies suitable for analysis. Another publication was incorporated after examining references from the extracted articles [23]. Consequently, our meta-analysis consisted of 18 articles (Fig. 1), including one case-control [20] and 17 cohort studies [10–19, 21–25, 32].

### Characteristics of included studies

Most studies were conducted in the United States and Europe, except one cohort study in Egypt [12], and were published between 1990 and 2016 (Table 1). Overall, the studies were well done, with a median score of 7 (range 6–8) on the Newcastle–Ottawa scale for appraising the quality of observational studies.

**Table 1:** Characteristics of studies included in the meta-analysis

Study	Year	Location	Research type	ICU type	NOS	Age, y (media)	Male/Femal	Period, month	Selected population	Sample	Detective way
Domart [25]	1990	France	CP	S	7	53	97/18	66	Mediastinistis after cardiac surgery	Blood, urine	Culture
Cook [24]	1998	USA	CR	S	7	NA	NA	14	Sepsis without bacteria or fungus	Blood, sputum	Culture
Kutza [23]	1998	Germany	CP	S	6	58	27/7	NA	Sepsis	Blood	PCR, pp65
Heininger [22]	2001	Germany	CP	S	7	68	35/21	12	Seropositive patients after major surgery and trauma	Blood, sputum	PCR, Culture
Cook [21]	2003	USA	CP	S	8	59	62/42	15	Sepsis, SICU stay>7d	Blood, sputum	Culture
Jaber [20]	2005	France	CC	M-S	6	NA	138/62	78	fever>72 h, without bacteria or fungus	Blood	pp65
von Muller [19]	2006	Germany	CP	M	7	NA	15/10	9	septic shock, ICU stay>7d	Blood	pp65
Limaye [18]	2008	USA	CP	M-S	8	52	73/47	24	Seropositive	Blood	PCR
Ziemann [17]	2008	Germany	CR	S	7	NA	33/66	33	ICU stay>13d	Blood	PCR
Chiche [16]	2009	France	CP	M	7	63	159/83	24	Mechanical ventilation16	Blood, sputum	PP65, Culture
Chilet [15]	2010	Spanish	CP	S	7	NA	37/16	13	Seropositive, ICU stay>5d	Blood, sputum	PCR
Bordes [14]	2011	France	CP	S	7	52	22/7	20	Seropositive, burn patients,	Blood	PCR
Heininger [13]	2011	Germany	CP	M-S	7	68	67/19	30	Seropositive, severe sepsis	Blood, sputum	PCR
Osman [12]	2014	Egypt	CP	M	7	59	29/22	3	Mechanical ventilation	Blood	PCR
Walton [11]	2014	France	CP	M-S	7	NA	305/255	48	Sepsis	Blood	PCR
Ong [10]	2015	New Zealand	CP	M	8	NA	196/110	24	ARDS, Mechanical ventilation>4d	Blood	PCR
Frantzeska [32]	2015	Greece	CP	M	7	63	51/29	24	Seropositive, mechanical ventilation	Blood	PCR
Ong [33]	2016	Netherlands	CP	M	7	NA	164/107	36	ARDS, Mechanical ventilation>4d	Blood	PCR

*CP* cohort prospective research, *CR* cohort and retrospective research, *CC* case-control research, *ARDS* acute respiratory distress syndrome, *PCR* polymerase chain reaction, *NA* not available, *M-S* medical-surgical, *S* surgical, *M* medical

A total of 2398 patients were included, having been admitted to the ICU for a variety of reasons, with a median age of 59 years. The median period of prospective studies was 24 months, ranging broadly from 3 to 78 months. All studies used CMV blood assays, and 6 studies also assayed sputum samples. Most studies indicated that the frequency of sample collection was once a week. In our analysis, the methods used to assess CMV infection were virus culture, pp65 antigen detection and PCR detection of CMV DNA in ten, three and two studies, respectively, and combinations of two diagnostic methods in the remaining three studies.

### CMV infection and outcome in immunocompetent patients

**Fig. 2:**
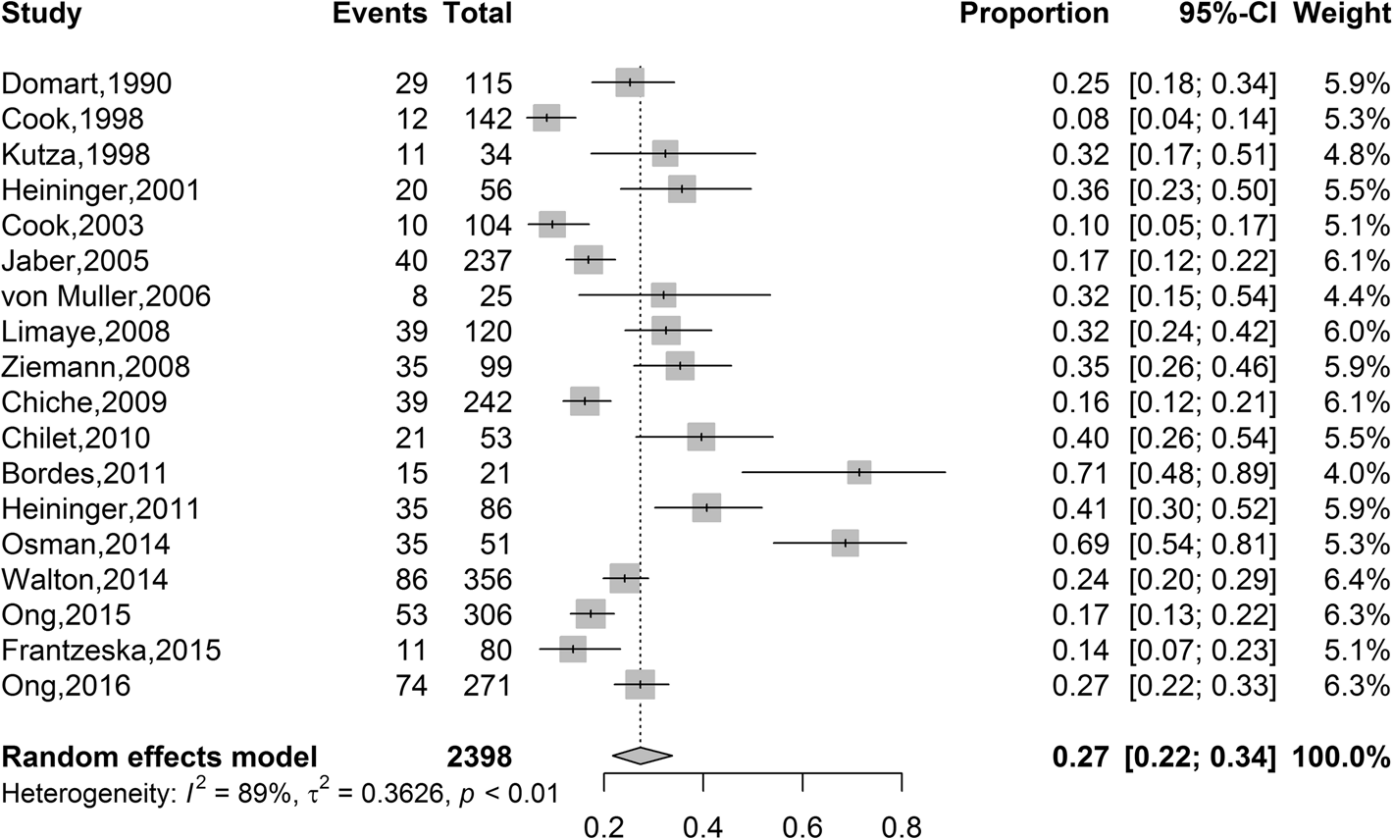
Overall detection rate of CMV

**Fig. 3:**
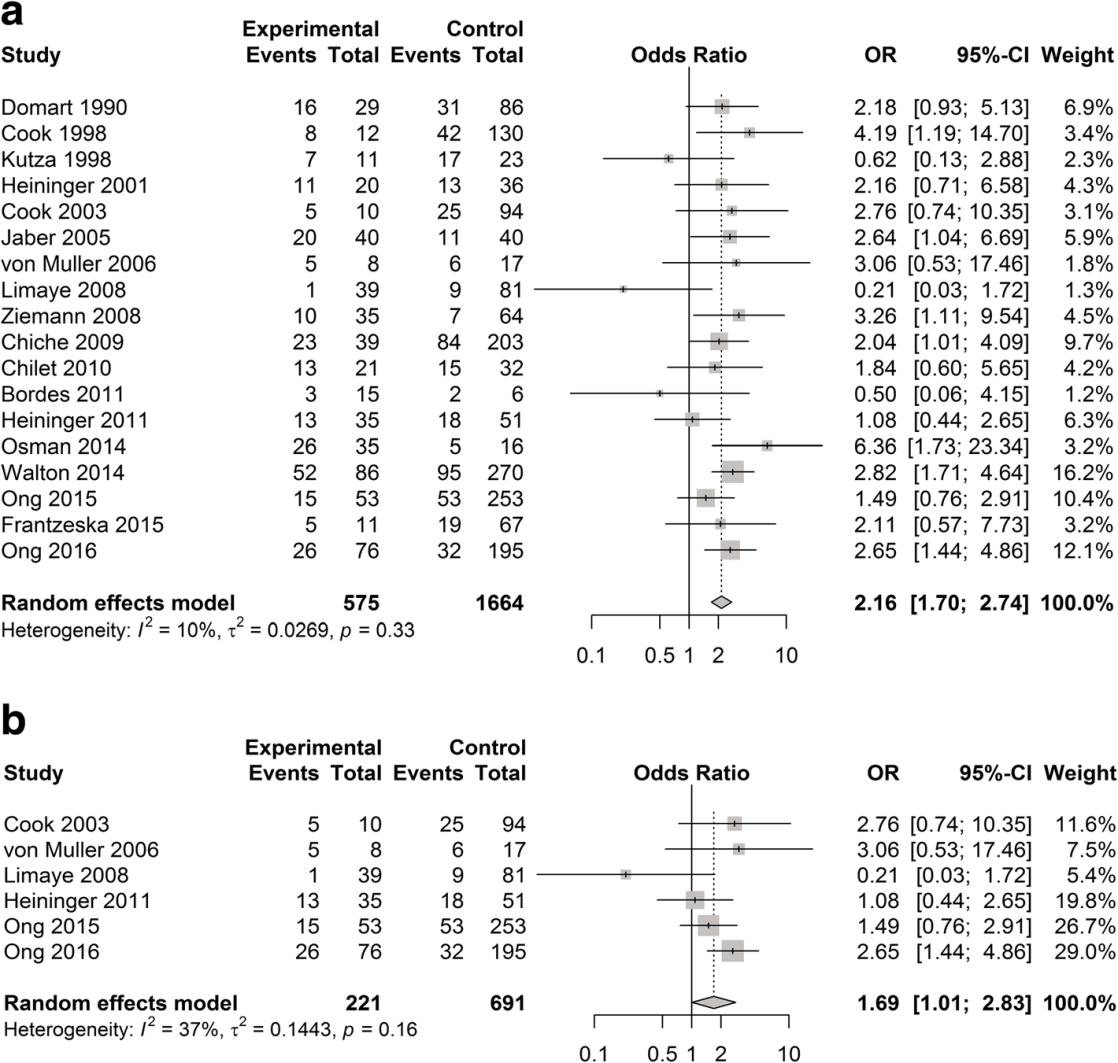
The effect of CMV infection on all-cause mortality in all trials(**a**), in patients without antiviral therapy(**b**)

As shown in Fig. 2, the overall detection rate of CMV was 27% (95% CI 22–34%, I^2^ = 89%, *n* = 2398). As compared with patients without CMV infection, the all-cause mortality of patients with CMV infection was significantly higher (OR: 2.16; 95% CI 1.70–2.74, I^2^ = 10%, *n* = 2239) (Fig. 3a). When analysis was limited to CMV detection in blood, there was still statistical significance in mortality rate between patients with CMV infection (OR: 2.15, 95% CI 1.48–3.15, I^2^ = 34%, *n* = 1441) compared with patients without infection (Additional file 2: Figure S1).

To rule out the impact of antiviral drugs on patients with CMV infection, four studies in which patients received antiviral drugs during their ICU stay and eight studies that did not specify the use of antiviral drugs were excluded. The remaining six studies of patients without antiviral treatment during the course of ICU stay were analyzed [10, 13, 18, 19, 21, 33]. The difference in mortality rates between patients with CMV infection remained significant (OR: 1.69, 95% CI 1.01–2.83, I^2^ = 37%, *n* = 912) compared with patients without infection (Fig. 3b). When analysis was limited to CMV detection in blood, there was no statistical significance in mortality rate between patients with CMV infection (OR: 1.69, 95% CI 0.81–3.54, I^2^ = 52%, *n* = 722) as compared with patients without infection (Additional file 3: Figure S2).

**Fig. 4:**
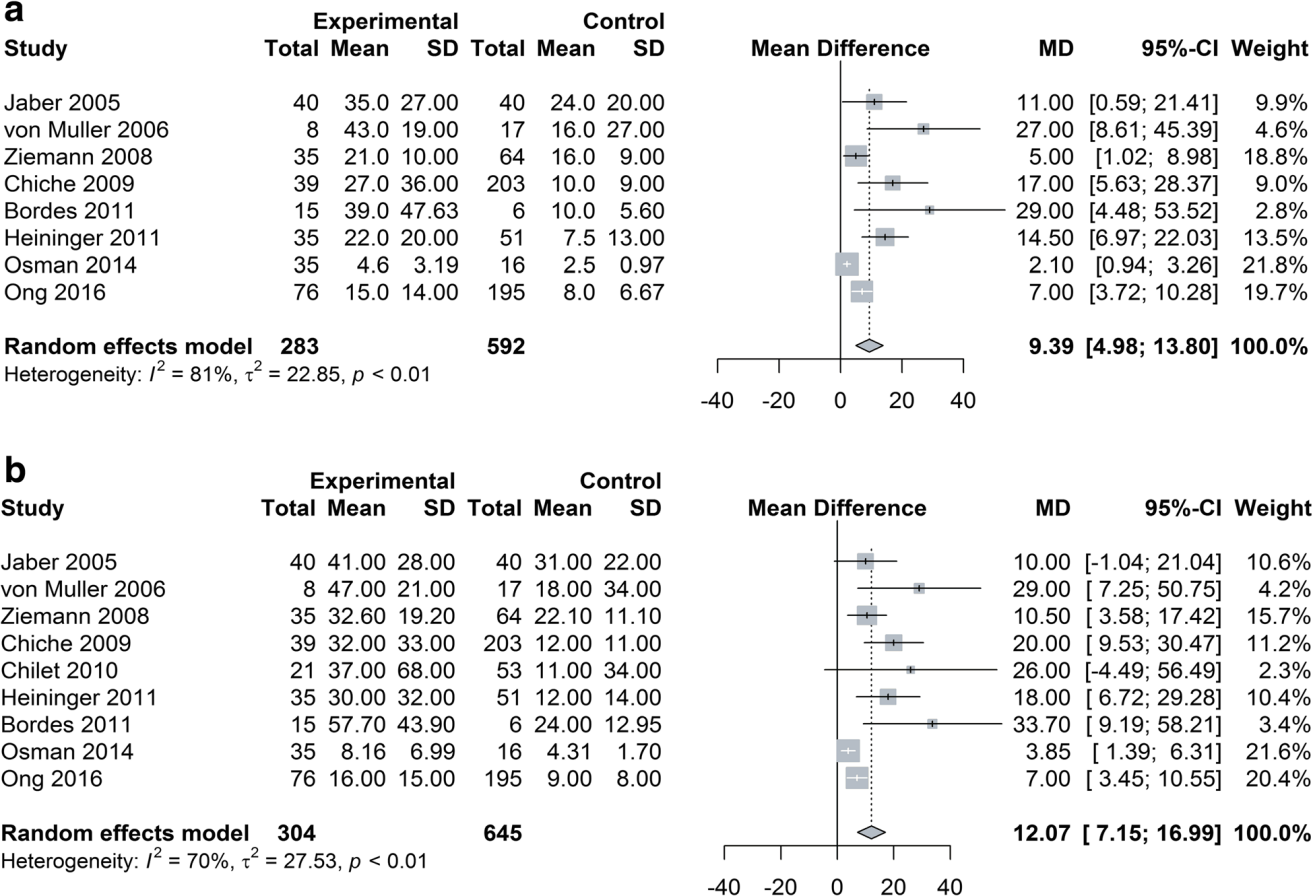
The mean difference in mechanical ventilation days in all trials (**a**) and the length of ICU stay in all trials (**b**) between active and non-CMV infection

The mean difference in mechanical ventilation days and duration of ICU stay was an increase of 9 days (95% CI 5–14, I^2^ = 81%, *n* = 875) and 12 days (95% CI 7–17, I^2^ = 70%, *n* = 949), respectively, between patients with and without CMV infection (Fig. 4a and b). When analysis was limited to CMV detection in blood, there was still a statistically significant difference in length of mechanical ventilation and ICU stay between patients with CMV infection as compared with patients without infection (MD: 7 days (95% CI 3–11, I^2^ = 77%, *n* = 547) and MD: 9 days (95% CI 4–13, I^2^ = 66%, n = 547)), respectively (Additional file 4: Figure s3 and Additional file 5: Figure s4).

### CMV reactivation and outcome in immunocompetent patients

**Fig. 5:**
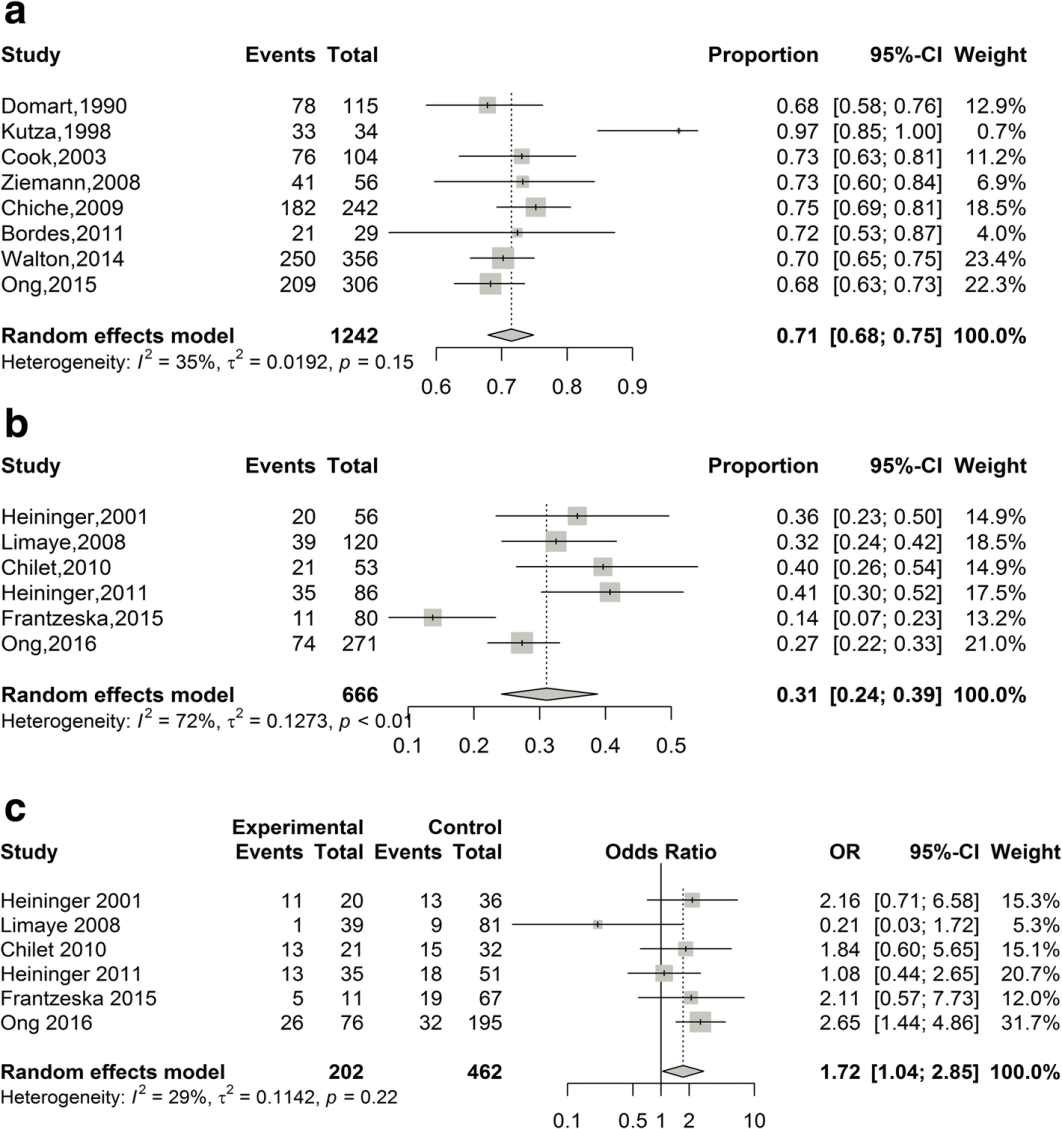
The rate of CMV seropositivity(**a**) and reactivation(**b**) and the forest plot displaying the effect of CMV reactivation on mortality in all trials(**c**)

The CMV seropositivity rate, which represents previous infection, was 71% (95% CI 68–75%, I^2^ = 35%, *n* = 1242) in immunocompetent ICU patients (Fig. 5a). Patients with CMV reactivation, which represents CMV detected among seropositive patients, was 31% (95% CI 24–39%, I^2^ = 72%, *n* = 666) (Fig. 5b).

The OR for mortality in patients with CMV reactivation as compared with patients without CMV reactivation was 1.72 (95% CI 1.04–2.85, I^2^ = 29%, *n* = 664) (Fig. 5c). But for patients of CMV infection in blood, the reactivation was not associated with higher mortality (OR: 1.49, 95% CI 0.46–4.28, I^2^ = 63%, *n* = 469) (Additional file 6: Figure S5).

We also analyzed the rate of CMV and mortality thought categorized by the detection methods (Additional file 7: Figure S6, Additional file 8: Figure S7: Additional file 9: Figure S8 and Additional file 10: Figure S9).

### Publication bias and sensitivity analysis

We used the Egger test to detect publication bias. There was no publication bias either in the overall CMV prevalence analysis (*t* = 1.1264, *p* = 0.2766) or in the all-cause CMV mortality analysis (*t* = − 1.3418, *p* = 0.1984). We also used Begg’s test to detect sensitivity analysis, and the results showed that the analyses were robust.

## Discussion

In this meta-analysis, we have demonstrated that CMV infection frequently present in critically ill immunocompetent patients at ICU admission. The overall rate of CMV infection was 27%, which was higher than the 17% presented in a previous meta-analysis [29], because eight recent studies detecting CMV infection by PCR assay were included in our meta-analysis [10–15, 32, 33]. Polymerase chain reaction has been demonstrated to be the most sensitive method of CMV detection [34], but even so, the CMV infection rate may still be underestimated because we chose only the studies containing 2 × 2 tables on CMV results and all-cause mortality. We excluded studies where either the rate of CMV infection or mortality was zero and we also excluded some studies with a 0% infection rate that used early monitoring of CMV, often fewer than 7 days after admission to the ICU [26, 35–37]. We believe this could have led to underestimation of the CMV infection rate because the transition to CMV infection requires time for the complete lytic virus cycle to develop from the latent phase [38].

We found that the detection rate of CMV by culture, pp65 and PCR was 13, 22 and 34%, respectively. Desachy et al. demonstrated that positive results for CMV infection were obtained in a median of 4 days by PCR compared with 11 days by pp65 antigen detection after onset of sepsis [36]. Therefore, PCR facilitates earlier diagnosis of an episode of CMV infection than any other method. We then analyzed the association between CMV positivity and mortality, stratified by detection method. We also found that patients with CMV infection detected by PCR had higher mortality than patients without CMV infection (OR: 2.07, 95% CI 1.59–2.70, I^2^ = 40%, *n* = 1441). However, when compared with other methods, the association with mortality was marginally less strong using PCR. We may think that viral burden of CMV is determinant of pathogenesis, and higher CMV loads is correlated with progression of some CMV infection disease [39, 40].

The presence of CMV seropositivity, representing previous infection, was found in 71% of immunocompetent ICU patients and the incidence of CMV reactivation was high, observed in 31% of seropositive patients in our meta-analysis. There are several factors that might explain the high prevalence. First of all, the rate of CMV seropositivity increases with advancing age [5] and in our analysis, the median age was 59 years. Second, to inhibit the reactivation of CMV, as many as 10% of all peripheral CD4+ and CD8+ T cells are constantly required for immune surveillance to maintain functional latency [41]. Sepsis is associated with immunoparalysis, as apoptosis of CD4+ and CD8+ T cells is increased [42, 43]. Furthermore, some patients in the ICU may be immunosuppressed after trauma and major surgery [44]. In addition, treatments commonly received in the ICU, such as massive transfusion, corticosteroids, or catecholamines may transiently compromise host immunity [45]. It has also been reported that the use of heart-lung machines can lead to temporary systemic immunosuppression [46]. Therefore, patients in the ICU may show transient immunoparalysis [47], potentially resulting in the observed CMV reactivation. Third, some inflammatory cytokines including tumor necrosis factor alpha and interleukin-1β, can stimulate reactivation of latent CMV [48]. Thus, significant numbers of immunocompetent patients harboring latent virus are susceptible to CMV reactivation during critical illness.

When the mortality analysis was limited to CMV detection in blood, CMV infection without antiviral drug treatment or reactivation was not significantly associated with higher mortality. This maybe explained that the presence of high peripheral levels of functional CMV-specific CD4+ and CD8+ T cells in immunocompetent patients, which can suppress CMV during episodes of reactivation [26]. It was observed that CMV infection was not associated with mortality in CMV colitis. In steroid-refractory patients with ulcerative colitis, CMV was found in the colon by histopathology, which is also not associated with adverse clinical outcomes [49]. Indeed, there has been no research to demonstrate that immunocompetent critically patients with CMV infection could benefit from antivirus therapy. And there are a number of side effects of antiviral drugs, such as hematologic complications (neutropenia, anemia and thrombocytopenia), renal dysfunction, mental disorders [50]. Therefore, giving antiviral drugs to critically ill patients should be considered cautiously in terms of advantage-disadvantage ratio. To address this issue, there are two ongoing, blinded, randomized placebo-controlled clinical trials of an antiviral drug with activity against CMV in critically ill patients in the ICU (NCT 01335932, NCT 02152358).

Patients with sepsis have the highest incidence of CMV infection [22]. Early in 1990’s, bacterial sepsis was considered to trigger CMV reactivation [26]. The reactivation associated with sepsis was consequence of inflammatory stimulation, transient immune compromise, and maybe involving some component of epigenetic regulation of viral DNA [26].

There are five limitations in this study. First, we observed large heterogeneity in many of our analyses. However, little or no heterogeneity was observed in the meta-analysis of mortality outcome. Second, most studies were not blind, thus reducing the reliability of the results. Third, lack of sufficient data on clinical parameters (eg: severity of illness, cause of ICU admission, comorbidity) meant that stratified analyses based on such clinical characteristics were not possible. Fourth, the definition of the state of CMV infection was inconsistent and maybe restrictive to capture the dynamics of CMV infection. As such, we could not conduct meta-analysis with outcome data and this is a major limitation of our meta-analysis. Finally, one [10] cannot discount the effect of unmeasured confounders given the observational nature of the body of evidence comprising this meta-analysis.

## Conclusions

Our findings suggests that there is a high incidence of CMV seropositivity and CMV infection in critically ill patients without immunosuppression. This study suggest that CMV infection without antiviral drug treatment or reactivation in critically ill patients is associated with increased mortality, and is not associated with mortality when CMV infection is detected in blood. Further research is necessary to determine the full role of CMV in this vulnerable patient demographic.

## Additional files

Additional file 1: Table S1. The Newcastle–Ottawa scale (PDF 17 kb)

Additional file 2: Figure S1. The effect of CMV infection on all-cause mortality in blood (TIFF 276 kb)

Additional file 3: Figure S2. The effect of CMV infection on all-cause mortality in patients without antiviral therapy in blood (TIFF 150 kb)

Additional file 4: Figure S3. The mean difference in mechanical ventilation days in blood between active and non-CMV infection (TIFF 217 kb)

Additional file 5: Figure S4. The mean difference in the length of ICU stay in blood between active and non-CMV infection (TIFF 222 kb)

Additional file 6: Figure S5. The effect of CMV reactivation on mortality in blood (TIFF 133 kb)

Additional file 7: Figure S6. Subgroup analysis of CMV detection rate according to detection method in all trials (TIFF 552 kb)

Additional file 8: Figure S7. Subgroup analysis of CMV detection rate according to detection method in blood (TIFF 393 kb)

Additional file 9: Figure S8. The effect of CMV infection on all-cause mortality in subgroup analysis according to detection method in all trials (TIFF 582 kb)

Additional file 10: Figure S9. The effect of CMV infection on all-cause mortality in subgroup analysis according to detection method in blood (TIFF 420 kb)

## Abbreviations

CD: Cluster of differentiation
CI: Confidence interval
CMV: Cytomegalovirus
DNA: Deoxyribonucleic acid
HHV: Human herpes virus
ICU: Intensive care unit
OR: Odds ratio
PCR: Polymerase chain reaction
