# Supplementary figures and images for: Cytomegalovirus infection and outcome in immunocompetent patients in the intensive care unit: a systematic review and meta-analysis
Source: BMC Infect Dis. 2018 Jun 28;18:289. doi: 10.1186/s12879-018-3195-5 (PMC6027797; doi:10.1186/s12879-018-3195-5)

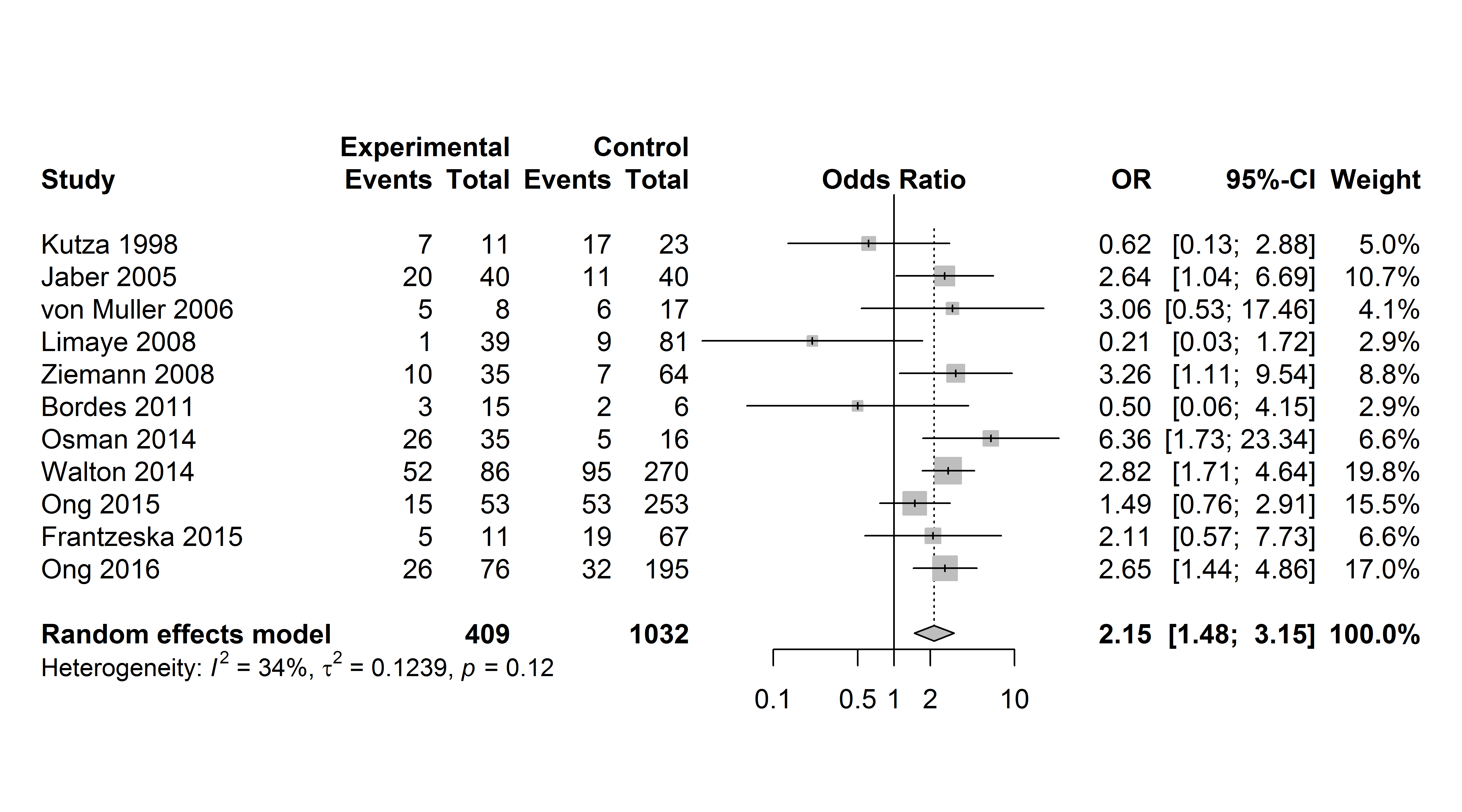

Supplement: Supplementary file 2 — Figure S1. The effect of CMV infection on all-cause mortality in blood (TIFF 276 kb) [file 12879_2018_3195_MOESM2_ESM.tiff]

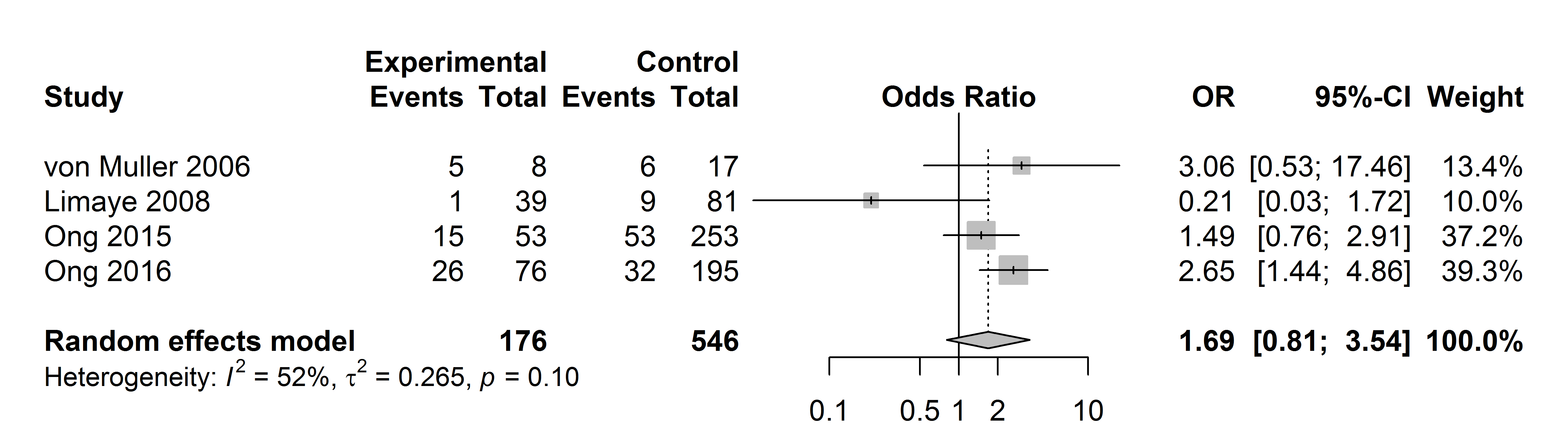

Supplement: Supplementary file 3 — Figure S2. The effect of CMV infection on all-cause mortality in patients without antiviral therapy in blood (TIFF 150 kb) [file 12879_2018_3195_MOESM3_ESM.tiff]

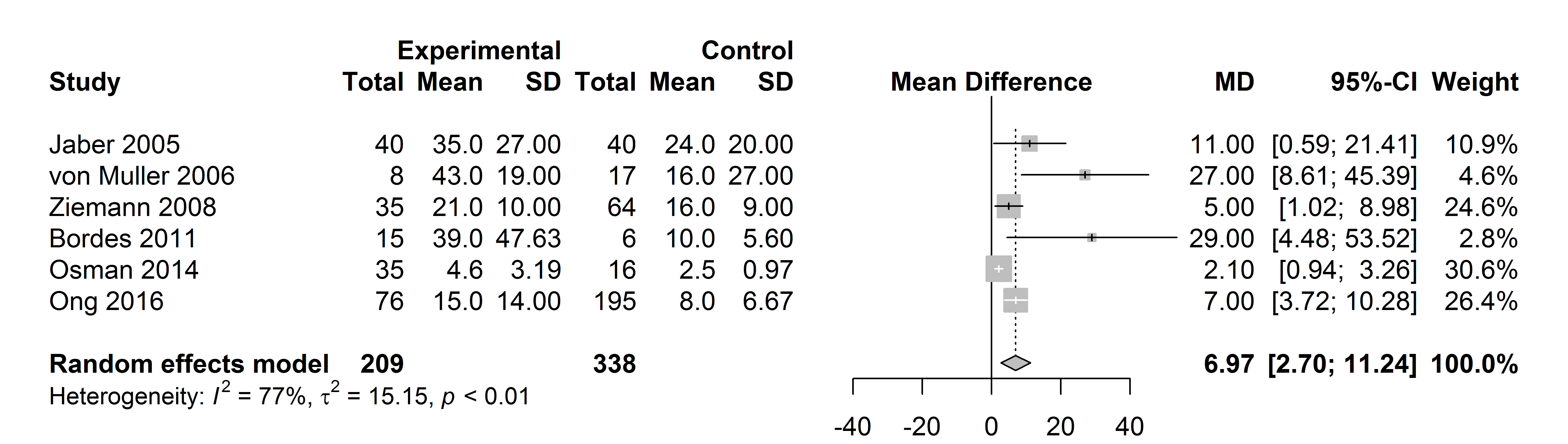

Supplement: Supplementary file 4 — Figure S3. The mean difference in mechanical ventilation days in blood between active and non-CMV infection (TIFF 217 kb) [file 12879_2018_3195_MOESM4_ESM.tiff]

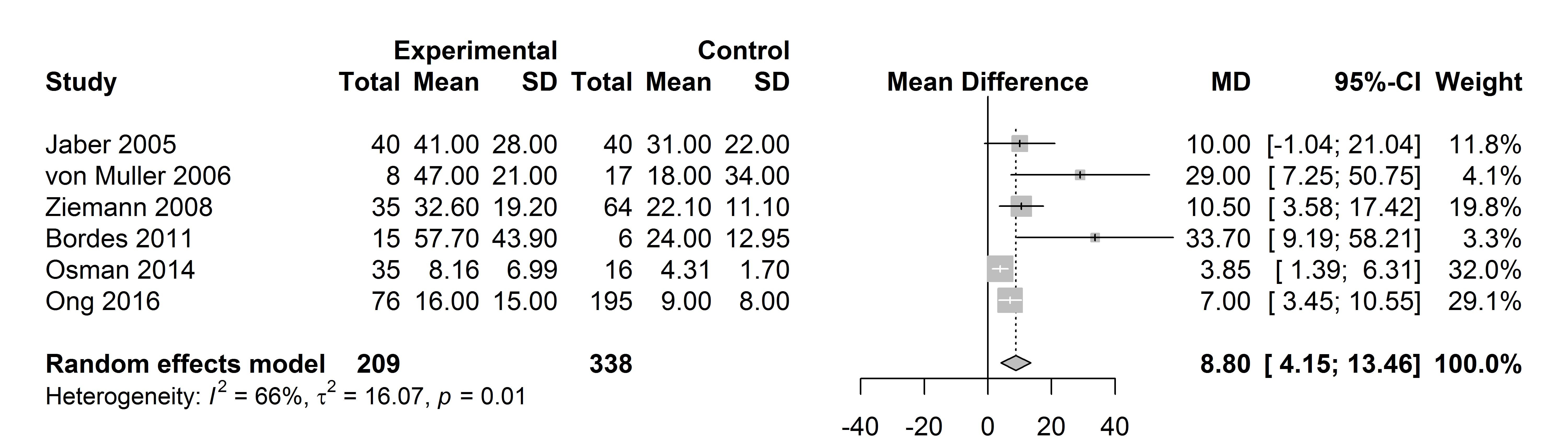

Supplement: Supplementary file 5 — Figure S4. The mean difference in the length of ICU stay in blood between active and non-CMV infection (TIFF 222 kb) [file 12879_2018_3195_MOESM5_ESM.tiff]

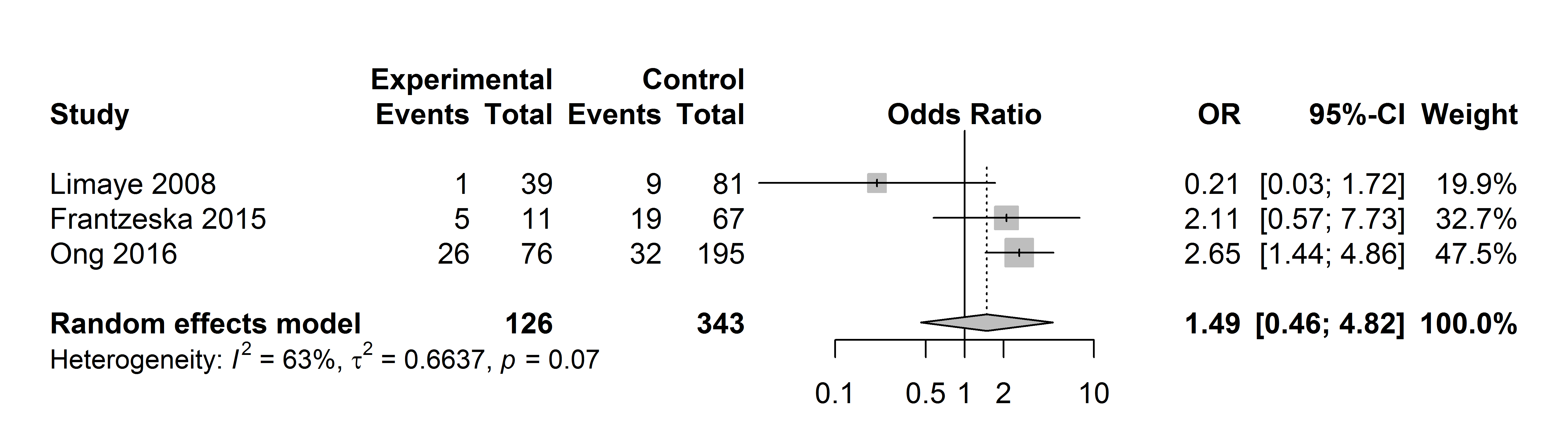

Supplement: Supplementary file 6 — Figure S5. The effect of CMV reactivation on mortality in blood (TIFF 133 kb) [file 12879_2018_3195_MOESM6_ESM.tiff]

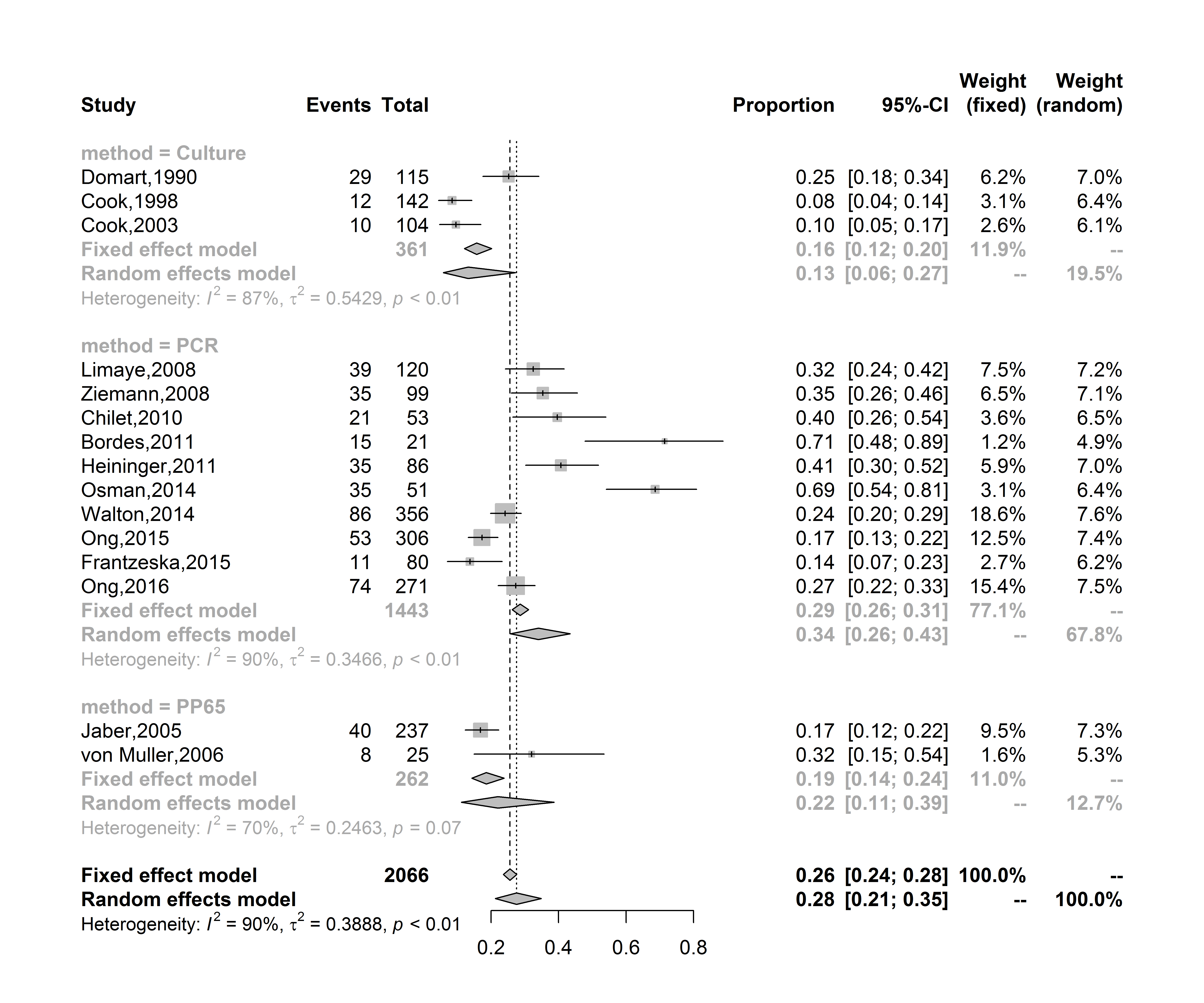

Supplement: Supplementary file 7 — Figure S6. Subgroup analysis of CMV detection rate according to detection method in all trials (TIFF 552 kb) [file 12879_2018_3195_MOESM7_ESM.tiff]

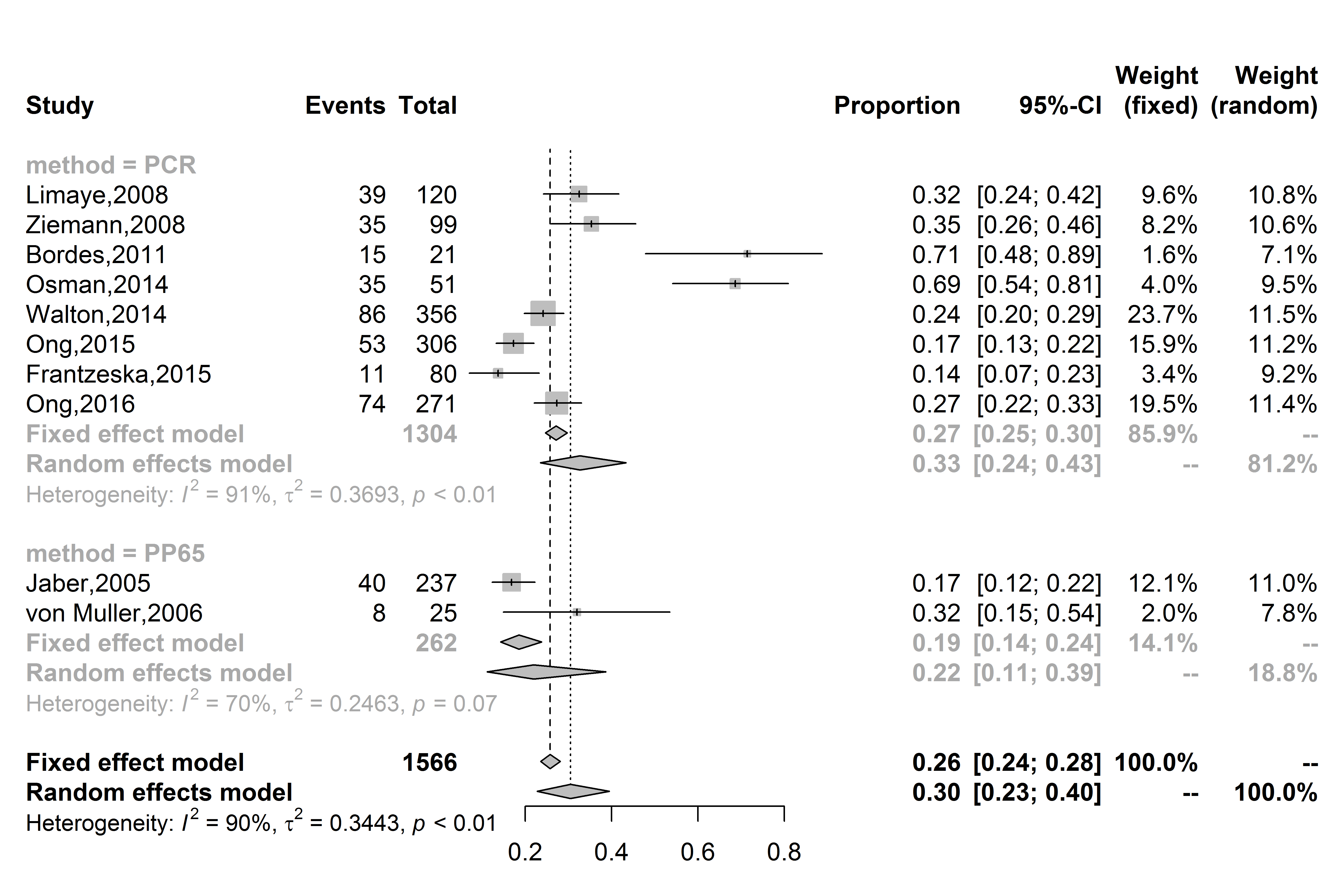

Supplement: Supplementary file 8 — Figure S7. Subgroup analysis of CMV detection rate according to detection method in blood (TIFF 393 kb) [file 12879_2018_3195_MOESM8_ESM.tiff]

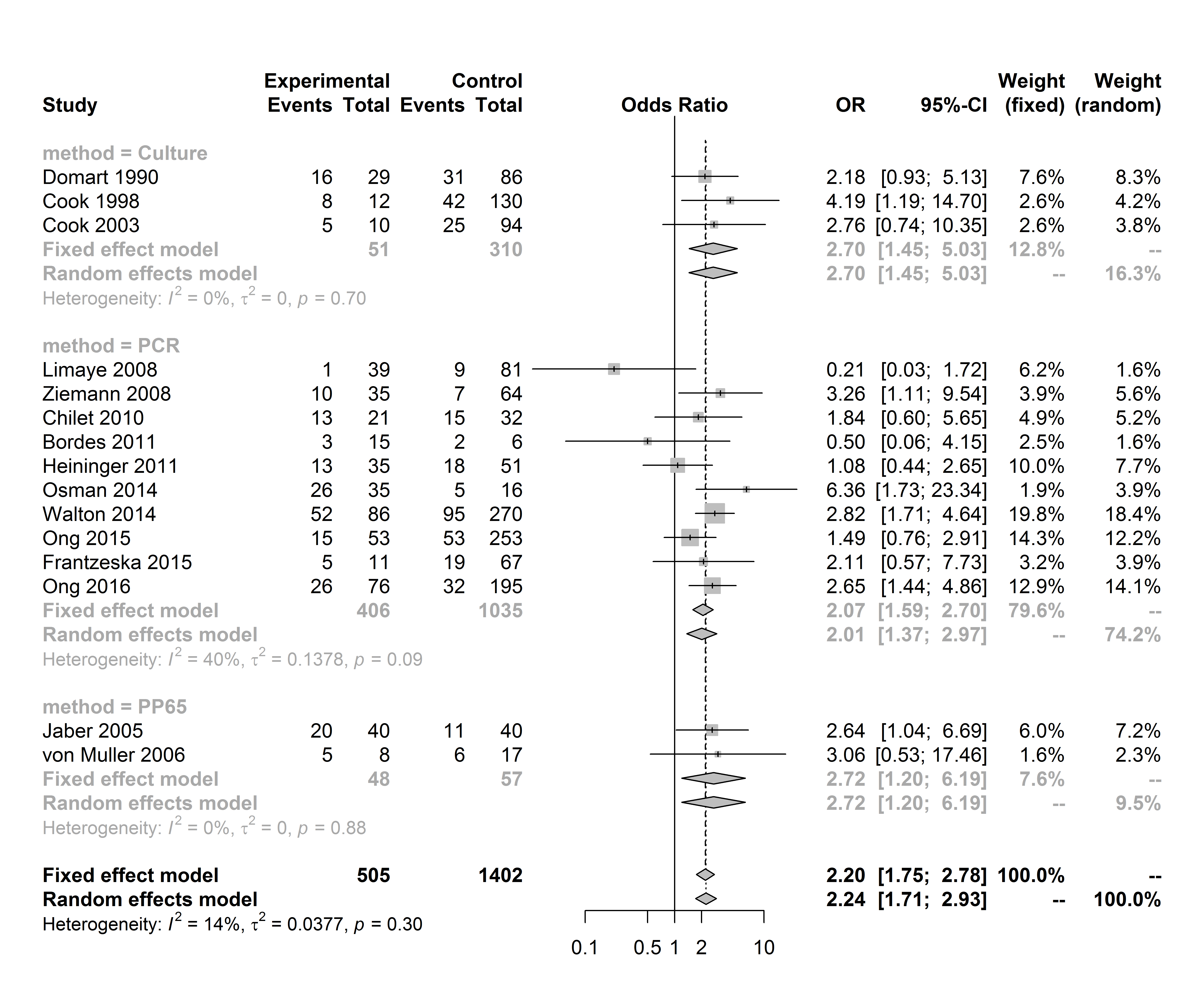

Supplement: Supplementary file 9 — Figure S8. The effect of CMV infection on all-cause mortality in subgroup analysis according to detection method in all trials (TIFF 582 kb) [file 12879_2018_3195_MOESM9_ESM.tiff]

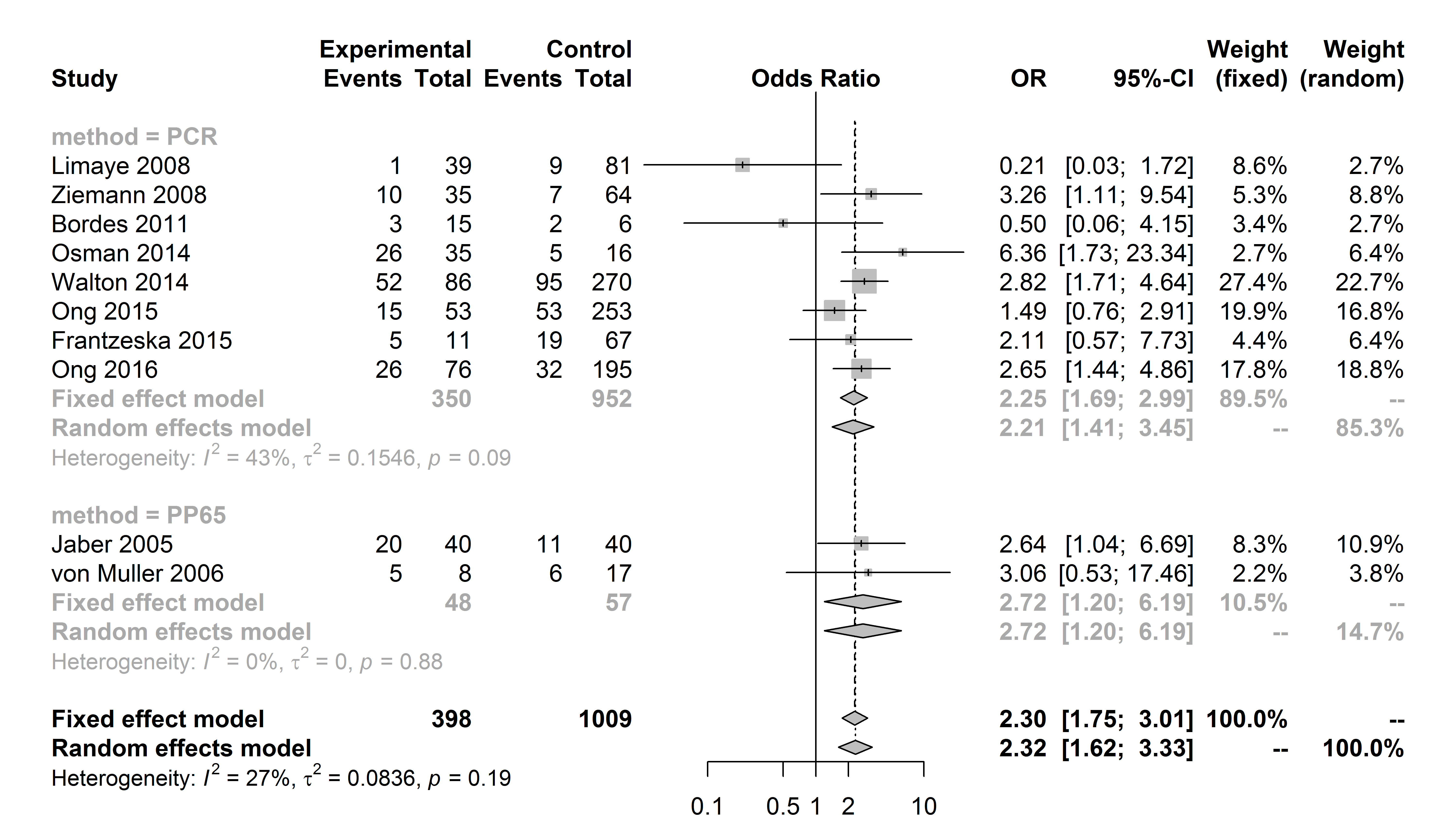

Supplement: Supplementary file 10 — Figure S9. The effect of CMV infection on all-cause mortality in subgroup analysis according to detection method in blood (TIFF 420 kb) [file 12879_2018_3195_MOESM10_ESM.tiff]
